# Introducing a novel respiratory function monitor for neonatal resuscitation training

**DOI:** 10.1016/j.resplu.2023.100535

**Published:** 2023-12-30

**Authors:** A.M. Dalley, K.A. Hodgson, J.A. Dawson, M.B. Tracy, P.G. Davis, M. Thio

**Affiliations:** a Newborn Research Centre, The Royal Women’s Hospital, Level 7, 20 Flemington Road, Parkville, Victoria 3052, Australia; b Department of Obstetrics, Gynaecology and Newborn Health, The University of Melbourne, Level 7, 20 Flemington Road, Parkville, Victoria 3052, Australia; c Paediatric Infant and Perinatal Emergency Retrieval, The Royal Children’s Hospital, 50 Flemington Road, Parkville, Victoria 3052, Australia; d Murdoch Children’s Research Institute, 50 Flemington Road, Parkville, Victoria 3052, Australia; e Westmead Hospital, Department of Neonatology, Cnr Hawkesbury Road and Darcy Road, Westmead, New South Wales 2145, Australia; f The University of Sydney, City Road Level 2 & 3, Sydney, New South Wales 2006, Australia; g Gandel Simulation Service, The Royal Women’s Hospital & The University of Melbourne, 20 Flemington Road, Parkville, Victoria 3052, Australia

**Keywords:** Respiratory function monitor, Face mask ventilation, Neonatal resuscitation, Resuscitation training, Resuscitation technology, Positive pressure ventilation

## Abstract

**Background:**

A respiratory function monitor (RFM) gives immediate feedback, allowing clinicians to adjust face mask ventilation to correct leak or inappropriate tidal volumes. We audited the satisfaction of clinicians with a neonatal resuscitation training package, incorporating a novel RFM.

**Methods:**

This was a mixed-methods study conducted at The Royal Women’s Hospital, Melbourne, Australia. Clinicians were approached to complete a neonatal resuscitation training session. Participants watched a training video, then provided ventilation to term and preterm manikins first without, and then with, the RFM. Clinicians completed a survey after the session and undertook a follow-up session three months later. The primary outcome was participant satisfaction with the RFM. Secondary outcomes included participants’ self-assessment of face mask leak and tidal volumes when using the RFM.

**Results:**

Fifty clinicians completed both the initial and follow-up session. Participants reported high levels of satisfaction with the RFM for both term and preterm manikins: on a scale from 0, meaning “not at all”, and 100, meaning “yes, for all resuscitations”, the median response (interquartile range, IQR) was 82 (74–94) vs 81.5 (69–94.5). Levels of satisfaction were similar for less experienced and more experienced clinicians: median (IQR) 83 (77–93) vs 81 (71.5–95) respectively. When using the monitor, clinicians accurately self-assessed that they achieved leak below 30% and tidal volumes within the target range at least 80% of the time.

**Conclusion:**

Clinicians of all experience levels had a high level of satisfaction with a training package including a novel RFM.

## Introduction

Although most infants manage the transition to independent breathing at birth, some require respiratory support to establish a functional residual capacity and initiate pulmonary gas exchange.^1^, ^2^ Ventilation via a facemask is the cornerstone of neonatal resuscitation^3^. In Australia in 2020, more than 9,000 infants (4% of live births) received positive pressure ventilation (PPV) with a facemask at birth^4^, ^5^. PPV is provided by a pressure generating device which delivers an unmeasured tidal volume (V_T_) to the lungs 2, 4, 6. A seal between a correctly sized mask and the infant’s face is required to deliver adequate and consistent V_T_. Mask ventilation is often ineffective, due to face mask leak and airway obstruction, and clinicians may be unaware that they are delivering insufficient V_T_^7^, ^8^, ^9^. Leak may occur due to an improperly positioned mask, incorrectly sized mask, inadequate and uneven pressure applied downward on the mask or inadequate jaw lift 10, 11. Schmölzer *et al.* reported a median face mask leak of 29% (range 16–63%) during mask ventilation of preterm infants in the delivery room (DR)^12^. Participants’ estimation of V_T_ delivery in this study were inaccurate. If face mask leak is large, the infant may not receive adequate V_T_ and the resuscitation may be compromised ^13^. If too large a V_T_ is delivered to the infant, volutrauma (overdistention of the lung) may result. If V_T_ is variable, airway collapse and reopening may contribute to lung inflammation in preterm infants 14, 15.

Respiratory function monitors (RFMs) provide a continuous display of mask leak and V_T_, allowing clinicians to correct their technique and maintain V_T_ in the target range of four to eight mL/kg 16, 17. While RFM use reduces face mask leak in manikin studies 18, 19, results from clinical trials have been conflicting: two studies reported a reduction in face mask leak, while the third and largest reported no difference in leak with an RFM visible 13, 16, 20, 21, 24. Randomised trials of RFMs in the DR have not been able to demonstrate consistent improvement in clinically important outcomes 20, 21.

**Fig. 1:**
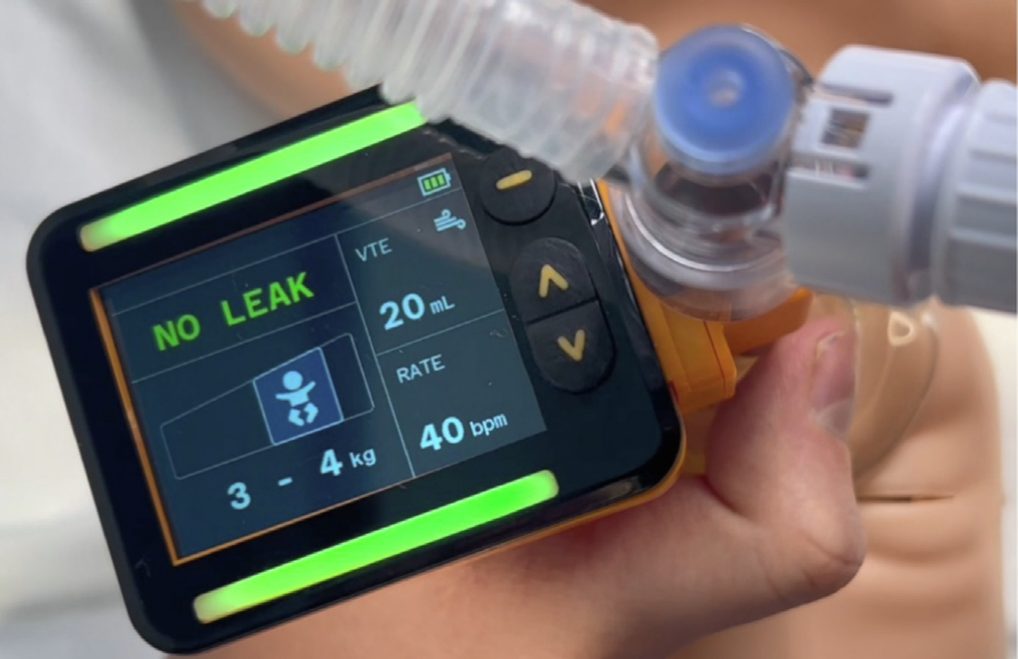
The Juno RFM (author’s own).

Systematic reviews have noted gaps in knowledge regarding human factors and training requirements 19, 20. Some currently available RFMs are bulky and are positioned at a distance from the infant. A newly developed small and portable RFM was used in this study (Fig. 1). It is placed directly above the face mask, giving clinicians feedback on leak and V_T_. The aim of this study was to audit the satisfaction of clinicians with a newly developed neonatal resuscitation training package incorporating this RFM.

## Methods

### Study design

This was a single-centre, mixed-methods study conducted in the Neonatal Intensive Care Unit at The Royal Women’s Hospital, Melbourne, Australia. Doctors and nurses with variable experience in neonatal resuscitation were eligible to participate. Two sessions were planned for each participant over a four-month period, to assess skill and knowledge retention. A convenience sample of 50 participants was enrolled. Clinicians completed the initial training session in May-June 2022, and a follow-up session three months later. This study was approved by the Royal Women’s Hospital Research Committee (AQA 22–10) as an observational audit of clinical practice. Participation implied consent to analyse the data.

### Training package and training session

A four-minute training video was developed demonstrating setup, use and interpretation of data from the novel RFM (video – supplementary content). Training sessions were run by a primary facilitator (responsible for introducing and leading session) with assistance from a secondary facilitator.

The training sessions were standardised: the facilitator explained the purpose of the session and demonstrated setup and interpretation of data from the RFM, participants were given unlimited time to practice using the RFM on a manikin (RFM set up, ventilation with T-Piece, interpretation of leak and V_T_, trialling multiple RFM positions and repositioning mask to reduce mask leak <30% while keeping V_T_ 4–8 mL/kg). Participants then provided 60 seconds of intermittent positive pressure ventilation (IPPV) using a face mask without the RFM, followed by 60 seconds with the RFM in situ and screen visible. Choice of mask hold was at the discretion of the individual clinician. This was first performed on a term manikin (Baby Anne term manikin, Laerdal, Stavanger, Norway), then repeated on a preterm manikin (Premature Anne manikin, Laerdal, Stavanger, Norway). Both manikins were leak free.

For the follow-up session, participants were given the option to watch the training video and there was no demonstration of setup and interpretation of data from the RFM. Participants were given time to practice using the RFM, then were asked to provide 60 seconds of IPPV using a face mask without the RFM, followed by 60 seconds with the RFM in situ and screen visible. This was performed on a term manikin first, then repeated on a preterm manikin.

### Equipment

Participants completed training sessions using the Juno Training Monitor (ResusRight™, Sydney, Australia) that measured airflow between the mask and T-piece device using a Micro Electromechanical Systems (MEMS) thermal flow sensor with a dead space of <1 mL and an accuracy of ±3% (Juno Sensor Specifications, Matt Crott, ResusRight™). The monitor automatically calculates and displays expiratory tidal volume (VTE) passing through the sensor with an accuracy of ±0.5 mL or ±8% and ventilation rate with an accuracy of ±1 ventilation per minute (Juno Training Monitor Product Information, Matt Boustred, ResusRight™). Clinicians used a T-piece device (Neopuff, Fisher & Paykel Healthcare, Auckland, New Zealand) to administer IPPV; default settings were a gas flow of 10 L/min, peak inspiratory pressure of 30 cm H_2_O for the term manikin and 25 cm H_2_O for the preterm manikin, positive end expiratory pressure of 5 cm H_2_O for both the term and preterm manikin. Clinicians used a size 0/1 60 mm diameter round silicone face mask (Laerdal, Stavanger, Norway) for the term manikin, and a 35 mm mask (Fisher & Paykel Healthcare, Auckland, New Zealand) for the preterm manikin.

### Outcome measures

The primary outcome was participant satisfaction with use of the RFM. This was evaluated by analysing clinicians’ responses to a survey created using a research electronic data capture (REDCap) tool hosted at Murdoch Children’s Research Institute 22, 23. Participants accessed the survey via a QR code and answered questions by selecting their response on a slider scale from 0 to 100. Secondary outcomes included participants’ self-assessment of face mask ventilation when using the RFM and participant satisfaction with the training package. Secondary outcomes were assessed by analysing data from the RFM and analysing survey responses. Data from the RFM were automatically saved as a CSV file displaying time since device boot (ms), inspiratory and expiratory tidal volume (mL), inspiratory and expiratory time (ms) for every given inflation detected and downloaded to a PC.

### Respiratory analysis

The following data were recorded on the monitor for 60 s period of IPPV on both term and preterm manikins: inspiratory and expiratory tidal volume (mL) and inspiratory and expiratory time (ms). Rate and leak were calculated from these data and displayed on the RFM screen. Prior to analysis of RFM data, leak was calculated in Excel (Microsoft®) using the following formula: (inspiratory V_T_-expiratory V_T_)/inspiratory V_T_ × 100.

### Statistical methods

Normally distributed data are presented using mean (SD) and student’s t-test was used for subgroup analysis. Skewed data are presented using median (IQR), a Mann Whitney U test was used for subgroup analyses (manikin size and operator experience). A *p* value less than 0.05 was considered statistically significant.

RFM data were cleaned to remove negative leaks and implausibly large tidal volumes (Table AI – supplementary content).

## Results

**Fig. 2:**
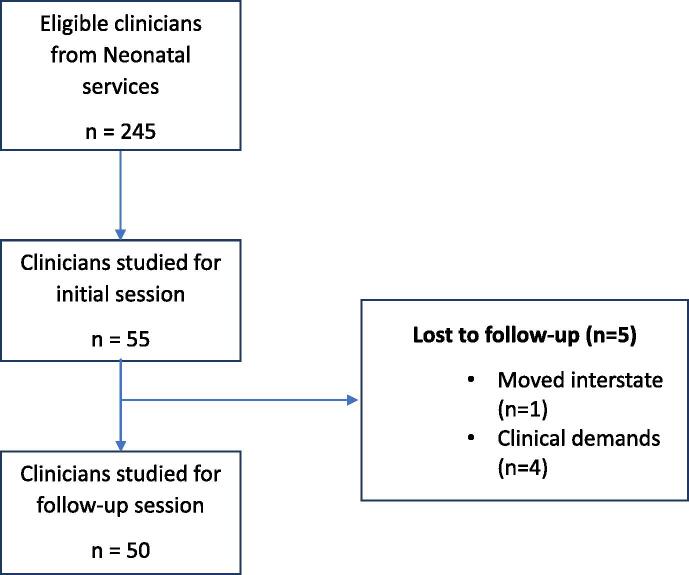
Recruitment flowchart for the NeoTrain study.

A total of 245 clinicians from Neonatal Services were eligible to participate in the study in May 2022, 55 were included in the initial training and 50 completed both an initial and follow-up training session (Fig. 2). Follow-up sessions were completed a mean (SD) of 11.2 (2.2) weeks after the initial session.

The 50 clinicians included in this study comprised 20 (40%) neonatal nurses, 14 (28%) registrars/residents, 8 (16%) neonatal fellows, 6 (12%) consultant (attending) neonatologists, and 2 (4%) neonatal nurse practitioners (Table 1). When surveyed after the initial training session, 26 (52%) clinicians reported having provided face mask ventilation >20 times in a clinical situation in the last year. Given the small numbers in each of the three groups with less mask ventilation experience (face mask ventilation 10–20, 5–10 and <5 times in the last year), the groups were combined for the analysis.

**Table 1:** Characteristics of participants included in the study (*n* = 50).

Participants	*n* = 50	*n* = 50
Participant position	*n* (%)	

Consultant	6 (12%)	
Fellow	8 (16%)	
Resident/registrar	14 (28%)	
Neonatal nurse practitioner	2 (4%)	
Neonatal nurse	20 (40%)	
Frequency of FMV in the last year	Initial training	Follow-up training

<5	10 (20%)	6 (12%)
5–10	7 (14%)	8 (16%)
10–20	7 (14%)	10 (20%)
>20 times	26 (52%)	26 (52%)
Previously used an RFM in the DR	7 (14%)	

FMV – face mask ventilation, RFM – respiratory function monitor.

Overall, clinicians consistently responded that they would find the RFM helpful in the DR during resuscitation (Table 2). Ratings of RFM helpfulness were similar for term and preterm manikins (Table 2) (median 82 [74–94] vs 81.5 [69–94.5] respectively; *p* = 0.52). Ratings were also consistent for less experienced and more experienced clinicians (median 83 [77–93] vs 81 [71.5–95] respectively; *p* = 0.54). Participant satisfaction with the training video shown at the beginning of the training was high and similar across clinicians with different experience levels (89 [80–97] for less experienced vs 93 [76–100] for more experienced clinicians; *p* = 0.49). Despite overall high satisfaction, some interference with mask application and hold, and obstruction of chest visibility with the RFM in situ was noted (Table AII – supplementary content).

**Table 2:** Participant satisfaction with RFM for face mask ventilation. Displayed by operator experience during initial (1) and follow-up (2) training for different manikin size. Differences between manikin size and operator experience were not statistically significant.

	Frequency of FMV in the last year			
	Less experienced clinician (*n* = 24)	More experienced clinician (*n* = 26)		
	Would you find this device helpful in the DR?			
	Response, median (IQR)	Response, median (IQR)	Total	*p* value
Term manikin(1)	85 (80–100)^a^	81.5 (72–90)	82 (76–95)	
Term manikin(2)	83 (77–89)^a^	78.5 (72–97)	82 (73–90)	
Total			82 (74–94)	
Preterm manikin(1)	81 (76–93.5)	83 (54–98)	81 (75–94)	
Preterm manikin(2)	81 (67.5–94.5)	84 (66–95)	82.5 (66–95)	
Total			81.5 (69–94.5)	
Total	83 (77–93)	81 (71.5–95)		0.54^b^
*p* value			0.52^c^	

Table shows responses to survey question: Would you find this device helpful in the DR when resuscitating a near term or term/very preterm newborn infant? (0 = Not at all, 100 = Yes, for all resuscitations).

RFM – respiratory function monitor, DR – delivery room, FMV – face mask ventilation, IQR – interquartile range.

a *n* = 1 missing survey response.

b Statistical analysis by clinician experience level (FMV ≤ 20 and >20 times in the last year).

c Statistical analysis by manikin size (term or preterm manikin).

Median (IQR) face-mask leak during the 60 s period when participants were ventilating using the RFM was 11% (3–22%) during the initial training and 16% (6–26%) during the follow-up session. Face mask leak was similar for both the term and preterm manikin (14% [7–23%] vs 11% [1–27%]; *p* = 0.55) across both the initial and follow-up training sessions. Leak > 60% was observed in 5% of all inflations, during initial and follow-up sessions. Low leak (<30%) was found in 84% of inflations during the initial sessions and in 81% of inflations during follow-up. The mean (SD) expiratory tidal volume (VTE) recorded during 60 s period when participants were ventilating using the RFM was within the clinical target range of 4–8 mL/kg. For the term manikin, less experienced clinicians had a higher mean (SD) VTE compared with more experienced clinicians (16.4 (3.9) mL vs 16.0 (4.1) mL; *p* = 0.08)**.** For the preterm manikin, less experienced clinicians had a lower mean (SD) VTE compared to those more experienced (5.2 (1.1) mL vs 5.3 (1.0) mL; *p* = 0.3).

**Fig. 3:**
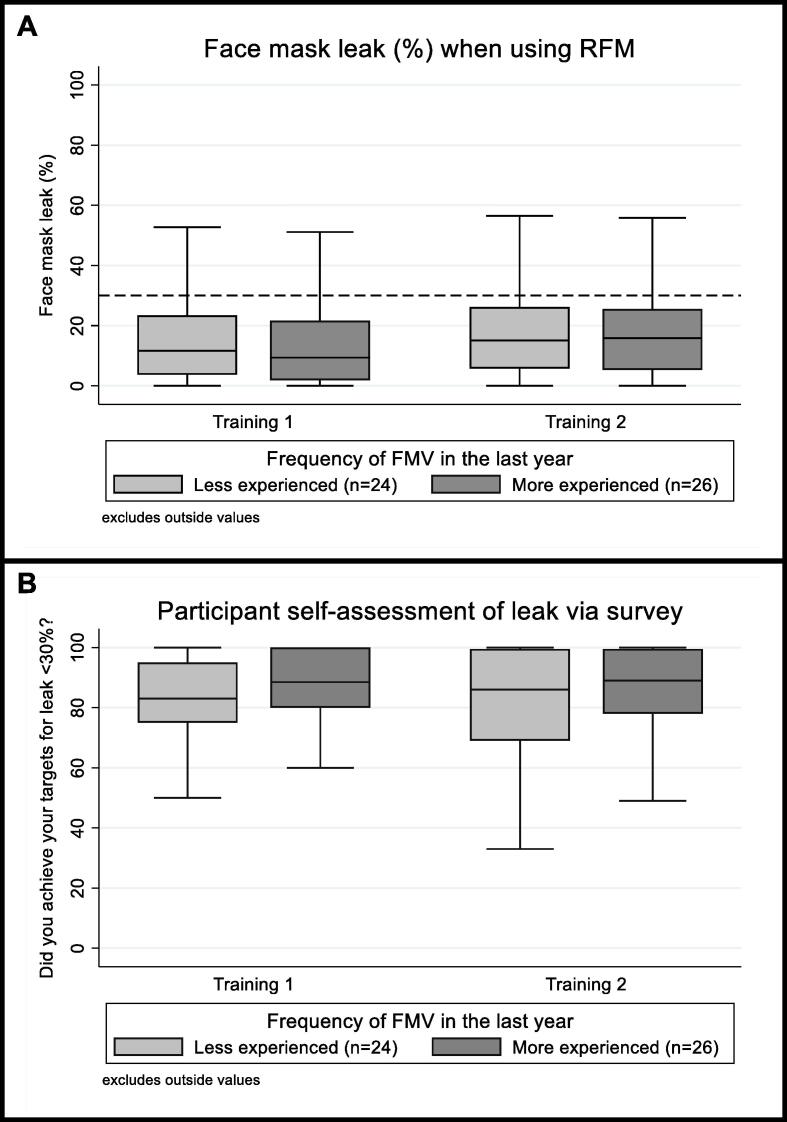
Boxplots comparing measurements of clinician’s face mask leak (%) during training sessions (A) to their self-assessment of leak targets via survey (B) for different experience levels (face mask ventilation (FMV) less than or equal to 20 (*n* = 24) or more than 20 (*n* = 26) times in the last year). The median is represented by the centre horizontal line of the box, the interquartile range (IQR) is shown by upper and lower edges of box and range is shown by the whiskers. <30% leak level is shown by the horizontal dashed line.

Fig. 3 shows the median mask leak measured for each experience level of face mask ventilation compared with self-assessment of leak targets via survey. When asked whether they achieved leak targets of <30% when using the RFM (0 = Never, 100 = Always), participants reported a median (IQR) response of 87 (76.5–99) in the initial training and a median (IQR) response of 87.5 (75–99.5) during the follow-up.

**Fig. 4:**
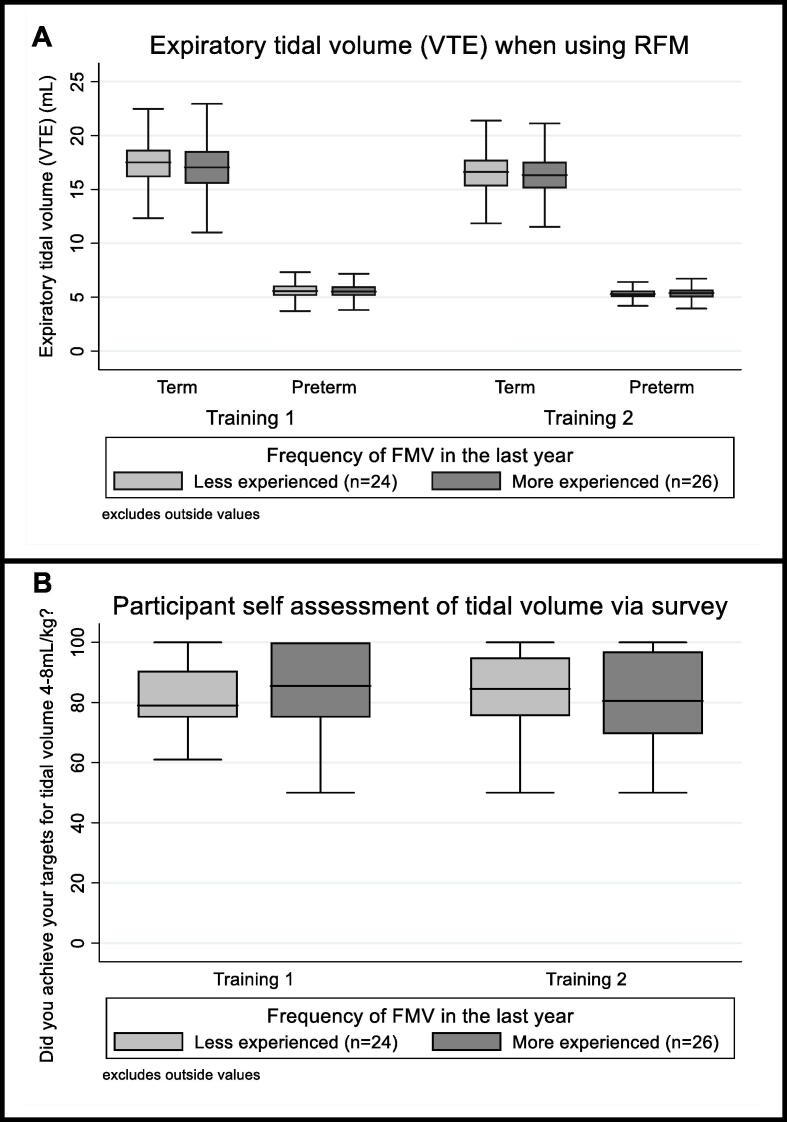
Boxplots showing measurements of clinicians expired tidal volume (mL) during training sessions (A) and their self-assessment of tidal volume targets via survey (B) for different experience levels (face mask ventilation (FMV) less than or equal to 20 (*n* = 24) or more than 20 (*n* = 26) times in the last year). The median is represented by the centre horizontal line of the box, the interquartile range (IQR) is shown by upper and lower edges of box and range is shown by the whiskers.

VTE was similar across both the initial and follow-up training (Fig. 4): mean (SD) 16.2 (4.0) mL for the term manikin and 5.3 (1.1) mL for the preterm manikin. These volumes fall within the clinical target range of 4–8 mL/kg for a term infant (i.e. weight 3,000–4,000 g) and an extreme preterm infant (i.e. weight 700–1000 g) respectively. When surveyed about whether they reached their target tidal volumes of 4–8 mL/kg (0 = Never, 100 = Always), participants correctly assessed that they mostly achieved their targets for tidal volume: median (IQR) response of 83% (75–93) of the time after the initial training and 84% (71–96.5) of the time after the follow-up training.

## Discussion

In this study, clinicians of all experience levels reported a high level of satisfaction with the novel RFM during both the initial and follow-up training sessions. This finding was consistent across preterm and term manikins and for less experienced and more experienced clinicians. When assessing face-mask ventilation competence while using the RFM, clinicians of all experience levels accurately estimated that they achieved leak below 30% and tidal volumes within the target range at least 81% of the time, despite reported interference with mask application and chest visibility.

Kuyper et al. surveyed clinicians using the New Life Box Neo-RSD RFM in a large multicentre trial in preterm infants 24, 25. They reported a similarly high level of satisfaction with the RFM: 99% of clinicians reported that the RFM was helpful during resuscitations; 92% reported that it influenced their decision making. However, 45% of these clinicians reported that they were insufficiently trained to use the RFM, and 78% reported that more training to interpret data from the RFM would be beneficial. Fifteen per cent of clinicians suggested the RFM should have a simpler visual interface.^25^

In this study, clinicians of all experience levels accurately estimated that they achieved leak below 30% and that they mostly achieved tidal volumes within the target range. The median mask leak of 11% with the RFM in situ during the initial training is similar to that reported by Wood et al. (mean leak of 11.2% during training using the Florian RFM) ^19^. Binder et al. used the Florian RFM during simulated neonatal cardiopulmonary resuscitation with external chest compressions and reported a median leak of 10% ^26^. The mean expiratory tidal volume (VTE) of 16.2 mL for the term manikin in our study was similar to that reported by O’Currain et al. with an RFM visible using a different term manikin (median VTE of 18.2 mL with RFM visible) ^18^. There are no studies that report VTE achieved using an RFM during training on a preterm manikin.

Schmӧlzer et al. reported that most clinicians were unable to accurately estimate the amount of face mask leak and VTE delivered to newborn infants during IPPV in the DR using clinical signs alone ^12^. In the simulation setting and with the aid of a novel RFM, clinicians across all levels of experience were able to accurately self-assess their ability to deliver tidal volumes within the target range and maintain minimum face mask leak across both the initial training and follow-up. Clinicians in our study may have been able to better self-assess their own performance during IPPV compared with previous studies because the novel RFM is a simpler device that displays less information and sits within the operator’s eyeline.

The strengths of our study are that we addressed both satisfaction with the RFM and the effect of using the RFM through measurements of face mask leak and tidal volume. Missing data were minimal and the follow-up rate for the repeat session was high (91%). The study sample included nurses and doctors of different experience levels, making the findings generalisable to staff of varying professional groups and experience.

However, findings from neonatal resuscitation studies on manikins cannot be translated directly into clinical practice. Mask ventilation is complicated by other factors when provided to a newborn infant in the DR, such as infant movement, surrounding activity of clinicians and other monitoring. Clinicians’ satisfaction with the novel RFM may differ in the clinical environment. Although a high proportion of eligible medical staff were recruited to this study (>75%), only 10% of eligible nurses were included. This may represent a bias.

One of the authors of this study is the Chief Clinical Advisor of ResusRight^TM^. To circumvent bias, he did not intervene in acquisition, analysis or interpretation of data.

### Future directions

A training format using the novel RFM may be developed to optimise (i) time required for clinician training and (ii) cost effectiveness. This may include groups of clinicians watching the training video together and using multiple RFMs simultaneously during the training session. Alternatively, this RFM may be used as an individual tool to maintain skills which could be accessed when clinical workload permits. Training costs include not only the cost of the novel RFM, but also the time and human resources required to train facilitators, the time added to a standard face-mask ventilation skills session for learners to practise, and other equipment including leak free manikins. The cost of the novel RFM training package, including the device, a carry case, charger, USB-C cable and one-year warranty, is approximately $950 US dollars. Our study showed high levels of satisfaction and accurate self-assessment of leak and V_T_ by trainees, in both trainings, when they had practised for a few minutes with the RFM device after watching the 5-minute training video. This implies learning does not take long and device knowledge can be retained. However, we observed that clinicians often benefited from the physical presence of an instructor to assist in adjusting mask hold or to remind them about RFM setup and interpretation of its data. Further studies are required to determine the effectiveness of the novel RFM in a self-training format. Future studies should address the use of this RFM during bag and mask ventilation, as this is the most common form of ventilation, particularly in low and middle-income countries. Finally, the effectiveness of simpler RFMs in the DR should be assessed in randomised clinical trials evaluating important clinical outcomes.

The novel RFM presents a flexible training model for clinicians who have variable and challenging clinical workloads. This study provides a strong rationale for future trials of the RFM in instructor based training and self-training of clinicians, as well as in the clinical environment.

## Conclusions

Clinicians of all experience levels had a high level of satisfaction with the RFM training package. Most clinicians were able to ascertain they were providing face mask ventilation with leak < 30% and a VTE within target for the hypothetical weights of the term and preterm manikins.

## Funding statement

This research did not receive any specific grant from funding agencies in the public, commercial, or not-for-profit sector.

## CRediT authorship contribution statement

**A.M. Dalley:** Investigation, Data curation, Formal analysis, Writing – original draft. **K.A. Hodgson:** Methodology, Investigation, Supervision, Writing – review & editing. **J.A. Dawson:** Methodology, Investigation, Supervision, Writing – review & editing. **M.B. Tracy:** Conceptualization, Writing – review & editing. **P.G. Davis:** Methodology, Supervision, Writing – review & editing. **M. Thio:** Conceptualization, Methodology, Investigation, Supervision, Writing – review & editing.

## Declaration of competing interest

The authors declare the following financial interests/personal relationships which may be considered as potential competing interests: ‘Co-author Dr Mark Tracy (Westmead Hospital and The University of Sydney) is the Chief Clinical Advisor to ResusRight™.’.
