# Supplementary material for: Introducing a novel respiratory function monitor for neonatal resuscitation training
Source: Resusc Plus. 2023 Dec 30;17:100535. doi: 10.1016/j.resplu.2023.100535 (PMC10792740; doi:10.1016/j.resplu.2023.100535)
Supplement: Supplementary data 1 [file mmc1.docx]

**Appendix A. Supplementary Content**

**Video A:** RFM training video, shown to participants at the beginning of training sessions.

**Table AI:** Cleaning actions taken for specific RFM data values and number of inflations discarded prior to analysis.

| RFM Data Values | Cleaning Action | | Cleaned or Discarded Data Values, n (%) |
| --- | --- | --- | --- |
| Negative leaks between 0 and -15% | Corrected to 0 leak | | Term  352 (7%)  Preterm  1231 (21%) |
| Negative leaks <-15% | Discarded |  | Term  50 (1%)  Preterm  109 (2%) |
| Term | Discarded | |  |
| V_T_ >35.17mL  (95th centile) |  |  | 262 (5%) |
| Preterm |  |  |  |
| V_T_ >19.16mL  (95th centile) |  |  | 297 (5%) |

Total of 306 inflations (5.9%) were discarded for the term manikin and 395 (6.7%) for the preterm manikin.

**Table AII:** Participant satisfaction with RFM for face mask ventilation: mask hold and chest visibility. Displayed by manikin size during initial (1) and follow-up (2) training for different manikin size. Differences were not statistically significant.

|  |  | **Response, median (IQR)** | |
| --- | --- | --- | --- |
|  |  | **Training 1** | **Training 2** |
| **Mask application difficult** | **Term**  **Preterm** | 24 (10-34)  41.5 (20-59) | 18 (9-39)  39 (14-64) |
| **Mask hold interference** | **Term**  **Preterm** | 25.5 (10-50)  31 (13-64) | 22 (10-50)  50 (17-65) |
| **Mask hold altered** | **Term**  **Preterm** | 23.5 (0-67)  50 (15-70) | 42.5 (12-66)  54.5 (17-79) |
| **Chest visibility** | **Term**  **Preterm** | 10.5 (0-24)  26 (7-50) | 10 (0-31)  20.5 (6-50) |

Table shows responses to survey questions:

Did the RFM make it more difficult to initially apply the mask correctly, as taught (0= Not difficult, 100= Very difficult)?

Did the RFM interfere with your normal mask hold during ventilation due to discomfort, weight or its shape (0= No, 100= Yes)?

Did you alter your mask hold when using the RFM (0= No, 100= Yes)?

Could you see the manikin’s chest whilst ventilating with the RFM in situ (0= Yes, clear view, 100= No, completely obstructed)?
